# Cooperation by Fibroblasts and Bone Marrow-Mesenchymal Stem Cells to Improve Pancreatic Rat-to-Mouse Islet Xenotransplantation

**DOI:** 10.1371/journal.pone.0073526

**Published:** 2013-08-29

**Authors:** Marcos Perez-Basterrechea, Alvaro J. Obaya, Alvaro Meana, Jesus Otero, Manuel M. Esteban

**Affiliations:** 1 Transplants, Cell therapy and Regenerative Medicine Unit, Hospital Universitario Central de Asturias, Oviedo, Spain; 2 Department of Functional Biology, Universidad de Oviedo, Oviedo, Spain; 3 CIBERER U714, Centro Comunitario de Sangre y Tejidos de Asturias, Oviedo, Spain; Université Paris Descartes, France

## Abstract

Experimental and clinical experiences highlight the need to review some aspects of islet transplantation, especially with regard to site of grafting and control of the immune response. The subcutaneous space could be a good alternative to liver but its sparse vasculature is its main limitation. Induction of graft tolerance by using cells with immunoregulatory properties is a promising approach to avoid graft rejection. Both Fibroblasts and Mesenchymal Stem Cells (MSCs) have shown pro-angiogenic and immunomodulatory properties. Transplantation of islets into the subcutaneous space using plasma as scaffold and supplemented with fibroblasts and/or Bone Marrow-MSCs could be a promising strategy to achieve a functional extra-hepatic islet graft, without using immunosuppressive drugs. Xenogenic rat islets, autologous fibroblasts and/or allogenic BM-MSCs, were mixed with plasma, and coagulation was induced to constitute a Plasma-based Scaffold containing Islets (PSI), which was transplanted subcutaneously both in immunodeficient and immunocompetent diabetic mice. In immunodeficient diabetic mice, PSI itself allowed hyperglycemia reversion temporarily, but the presence of pro-angiogenic cells (fibroblasts or BM-MSCs) within PSI was necessary to improve graft re-vascularization and, thus, consistently maintain normoglycemia. In immunocompetent diabetic mice, only PSI containing BM-MSCs, but not those containing fibroblasts, normalized glycemia lasting up to one week after transplantation. Interestingly, when PSI contained both fibroblasts and BM-MSCs, the normoglycemia period showed an increase of 4-times with a physiological-like response in functional tests. Histology of immunocompetent mice showed an attenuation of the immune response in those grafts with BM-MSCs, which was improved by co-transplantation with fibroblasts, since they increased BM-MSC survival. In summary, fibroblasts and BM-MSCs showed similar pro-angiogenic properties in this model of islet xenotransplantation, whereas only BM-MSCs exerted an immunomodulatory effect, which was improved by the presence of fibroblasts. These results suggest that cooperation of different cell types with islets will be required to achieve a long-term functional graft.

## Introduction

The current standard therapy for type 1 diabetes mellitus fails to achieve physiological control of blood glucose, increasing the risk of long-term diabetic complications. Pancreatic islet transplantation could be one of the alternatives to treat definitively type 1 diabetes, nevertheless, current protocols of clinical islet transplantation have not yielded long-term insulin-independence [1]. The experimental and clinical experiences highlight the need to review some aspects of islet transplantation protocol, especially with respect to the site of transplantation and the control of the intrinsic immune response [2].

The liver has been chosen as the reception site in clinical islet transplantation. However, experimental and clinical studies have shown several factors that indicate that the liver seems not to be the optimal place for islet grafts [3–5]. For this reason, alternative sites have been proposed such as spleen, renal capsule, testes, brain, peritoneal cavity, omentum, bone marrow, muscle, epididymal fat or the subcutaneous space [6–8]. The subcutaneous space constitutes a very attractive site of transplantation because it would provide a simple and safe procedure. Nevertheless, the sparse vasculature of this tissue restricts blood supply to the graft, which becomes essential for both islet survival and function during the first days after transplantation [9]. In addition, blood supply, is also especially important for restoring islet-extracellular matrix interactions affected by the islet isolation procedure [10,11]. Most of the attempts to overcome these great limitations have focused on the use of different biomaterials as scaffolds either alone or in combination with pro-angiogenic factors [12–15].

Control of the immune response has been tested in several approaches, with or without immunosuppressive drugs. Diffusion chambers or micro and macrocapsules, are devices that have been tried, wit the aim of providing enough oxygen flow while maintaining a barrier to immune cells [16]. Induction of graft tolerance by using cells with immunoregulatory properties is a promising approach to avoid graft rejection that has emerged in recent years. The beneficial effect of multipotent mesenchymal stem cells (MSCs) in transplant approaches has been recently attributed to its capacity in producing several factors with paracrine immunomodulatory properties [17,18]. Through secretion of these factors, mainly transforming growth factor (TGF)-β1, hepatic growth factor (HGF), nitric oxide, indoleamine 2,3-dioxygenase (IDO), prostaglandin E-2 (PGE-2), matrix metalloproteinase (MMP) -2 and -9 or interleukin (IL) -6, MSCs they are able to regulate both innate and adaptive immune response, controlling proliferation, migration and function of both B, T and NK cells, as well as inhibiting immunoglobulin and antibody production, dendritic cells maturation and neutrophils activation [19]. These cells have being used in clinical trials of “graft versus host disease” and autoimmune disease treatment with promising results [20] and are being evaluated in solid organ transplantation phase I/II clinical trials [21]. Regarding fibroblasts, direct comparison between adult fibroblasts from various tissues and bone marrow MSC showed similar *in vitro* immunosuppressive potency [22–24].

We have recently published a novel model of subcutaneous islet transplantation using a biodegradable plasma-based scaffold containing fibroblasts allowing long time graft survival in immunodeficient mice [25]. Taking into account their described properties, we explored whether the presence of autologous fibroblasts and/or allogenic BM-MSCs in our Plasma-based Scaffold containing Islets (PSI) could induce graft tolerance after subcutaneous transplantation in an immunocompetent recipient, improving graft viability, without using immunosuppressive drugs.

## Materials and Methods

### Animals and Ethics Statement

Twelve-week-old male Wistar rats (Animal Facilities, University of Oviedo, Spain) were used as pancreatic islet donors. Six-week-old male Swiss nu/nu mice (Harlan Iberica, Barcelona, Spain) were used as immunodeficient recipients. Six-week-old male mice from the commercial luminescent strain FVB/N-Tg (β-Actin-luc)-Xen mice (Caliper Life Sciences, Hopkinton, MA) were used as source of luminescent (Luc[+]) cells (fibroblasts or BM-MSCs). Six-week-old male non-bioluminescent (Luc[-]) mice from the FVB/N-Tg (β-Actin-luc)-Xen mice strain (Caliper Life Sciences) were used as immunocompetent recipients. Mice were rendered diabetic by a single intraperitoneal injection of streptozotocin (STZ) (225 mg/kg body weight; Sigma-Aldrich, Madrid, Spain). Diabetes was defined as non-fasting blood glucose (NFBG) levels higher than 450 mg/dL determined in two consecutive days [26]. Experiments were conducted in accordance with the guidelines of the European Union (86/609/EU) and Spanish regulations (BOE 67/8509-12, 1988). Experimental protocols were also approved by the Committee for Animal Care and Handling of the University of Oviedo (Permit number: 9-INV-2004). All surgery was performed under xylazine-ketamine anesthesia and animals were sacrificed using CO_2_. All efforts were made to minimize animal suffering and to reduce the number of animals used.

### PSI preparation and transplantation

#### Plasma

Blood was obtained from Wistar rats by venipuncture into 5-mL sodium citrate-coated sterile tubes (Vacutainer SST^™^ II Advance; Becton-Dickinson, Plymouth, United Kingdom) and centrifuged at 3000 rpm for 15 min. The plasma was collected and frozen until use.

*Pancreatic islets* were obtained as previously described [27]. Wistar rat pancreata were distended with an intraductal injection of collagenase type XI (Sigma-Aldrich). The pancreata were then harvested, incubated in a water bath at 37 ^°^C for 21 min, and the digested tissue washed in Hank’s balanced salt solution (HBSS, Sigma-Aldrich). Islets were separated by Dextran (Sigma-Aldrich) discontinuous density gradient centrifugation, washed in HBSS, and immediately prepared for transplant. Islet equivalents (IEQs), purity, and viability were calculated by examining dithizone-stained (Sigma-Aldrich) preparations.

*Skin fibroblasts* were obtained autologously (from nu/nu or FVB/N-Tg (β-Actin-luc)-Xen mice) as previously described [25]. Full-thickness skin biopsy specimen (1cm^2^) from the right flank was ground without previous dermis-epidermis separation, and the homogenate subjected to enzyme digestion with 2 mg/mL collagenase (Sigma-Aldrich) at 37 ^°^C for 4 h. The digested tissue suspension was then filtered through a cell strainer (Becton Dickinson) and centrifuged at 1400 rpm for 10 min. The resultant pellet was resuspended in Dulbecco’s modified Eagle’s medium (DMEM, Lonza, Basel, Switzerland) supplemented with 10% fetal calf serum (Gibco Invitrogen, Paisley, United Kingdom) and Antibiotic-Antimycotic solution (50 U/ml penicillin + 50 µg/ml streptomycin, Gibco Invitrogen), and cells were seeded in culture flasks and cultured at 37 ^°^C in a humidified atmosphere of 5% CO_2_. Fibroblasts were used after 4–5 passages.

#### BM-MSCs

Isolation of MSCs from nu/nu (immunodeficient experiments) or FVB/N-Tg (β-Actin-luc)-Xen mice (immunocompetent experiments) was carried out according to Soleimani M. et al [28] with some modifications. Briefly, bone marrow was collected by flushing femurs and tibias with complete medium: DMEM/Ham’s F12 medium (Lonza) + 20% fetal calf serum + Antibiotic-Antimycotic solution (50 U/ml penicillin + 50 µg/ml streptomycin, Gibco Invitrogen), supplemented with 5 U/mL heparin (Hospira Prod. Farm. SL, Madrid, Spain). Bone marrow cells were washed once with PBS, resuspended in complete medium and seeded in culture flasks to be cultured at 37 ^°^C in a humidified atmosphere of 5% CO_2_. BM-MSCs were used after 4-5 passages. In order to check pluripotency maintenance of these cells, osteogenic and adipogenic differentiation were verified by alkalyne phosphatase and oil red o staining, respectively (data not shown).

#### PSI preparation

Cultured cells (fibroblasts, BM-MSCs or both) were resuspended in plasma containing 5 mg/mL of tranexamid acid (Amchafibrin, Fides-Ecofarm, Barcelona, Spain). Next, 1500 fresh IEQs, obtained from two donor rats, were added to the mixture and the final volume was adjusted to 4 mL with DMEM + 6.75 mM CaCl_2_. The mixture was finally allowed to solidify for 15-20 minutes. Purity and viability of the islet samples used were not significantly different between the experimental groups (data not show).

#### PSI transplantation

Transplantation was performed in diabetic mice 7-10 days after STZ injection. Mean pre-transplant NFBG of mice, as well as purity and viability of the islet samples used, were similar in all groups. PSI was placed directly over the abdominal musculature after performing a skin incision. NFBG was monitored periodically after transplantation on whole blood samples obtained from the tail vein. Normoglycemia was defined as a NFBG level <200 mg/dL.

### Experimental groups

Unless mentioned, five animals were used in each experimental group.

#### Immunodeficient model

For graft function studies, the following groups were used: diabetic mice transplanted with free islets (ISC), PSI without cells (PSI), PSI containing 2x10^5^ autologous fibroblasts (PSI-F), PSI containing 2x10^5^ allogenic BM-MSCs (PSI-M) and PSI containing 2x10^5^ of both autologous fibroblasts and allogenic BM-MCSs (PSI-FM), as well as non-transplanted diabetic animals and non-transplanted healthy animals.

For cell evolution studies by bioluminescence follow-up, the following groups were performed: diabetic mice transplanted with PSI containing 10^6^ allogenic Luc[+]-fibroblasts (PSI-5F_luc_), PSI containing 10^6^ allogenic Luc[+]-BM-MSCs (PSI-5M_luc_) or PSI containing 10^6^ of mixed autologous Luc[-]-fibroblasts and allogenic Luc[+]-BM-MSCs (PSI-5FM_luc_).

#### Immunocompetent model

For graft function studies, the following groups were used: diabetic mice transplanted with free islets (ISC), PSI without cells (PSI), PSI containing autologous fibroblasts (PSI-F), PSI containing allogenic BM-MSCs (PSI-M), or PSI containing mixed autologous fibroblasts and allogenic BM-MSCs (PSI-FM), as well as non-transplanted diabetic animals and non-transplanted healthy animals. Three different numbers of cells were added to PSI: 2x10^5^, 4x10^5^ and 10^6^ of each cell type.

For cell evolution studies by bioluminescence follow-up, the following groups were performed: diabetic mice transplanted with PSI containing 10^6^ allogenic Luc[+]-BM-MSCs (PSI-5M_luc_) or with PSI containing 10^6^ of mixed autologous Luc[-]-fibroblasts and allogenic Luc[+]-BM-MSCs (PSI-5FM_luc_).

### Intraperitoneal glucose tolerance tests (IPGTTs)

After an overnight fast, the animals received an intraperitoneal glucose bolus (2 g/Kg of body weight) as a 5% solution in Hanks’ Balanced Salt Solution (Lonza), and blood glucose was monitored at 0, 15, 30, 60, 90 and 120 min. To evaluate the response to IPGTTs, the “area under the curve” (AUC) was calculated for each animal using the trapezoidal rule method [29].

### In vivo bioluminescence imaging

To monitor regularly the luminescence of Luc[+]-BM-MSCs or Luc[+]-fibroblasts subcutaneously transplanted in mice, the IVIS® Lumina (Caliper Life Sciences) *in vivo* noninvasive bioluminescence imaging system was used after an intraperitoneal injection of 150 mg/Kg D-Luciferin (Melford, Suffolk, United Kingdom) in PBS. Bioluminescence signal was measured using Living Image® software and normalized to the day of transplantation.

### Histology

In order to analyze histologically the transplanted xenografts, mice were sacrificed sixty (immunodefficient) or seven (immunocompetent) days after transplantation. Islet grafts were fixed in 4% paraformaldehyde for 24 h. After fixation, the specimens were dehydrated in a graded series of ethanol dilutions and embedded in paraffin. Sections of 4-µm thick were obtained using a microtome HM 350 S (Microm, Waldorf, Germany). Some sections were stained with hematoxylin and eosin (H&E) for microscopy examination. The presence of functional islets was confirmed by immunohistochemical insulin labeling. Briefly, paraffin sections were deparaffinized and rehydrated in EZ Prep, and placed in Reaction Buffer. The primary antibody was polyclonal Guinea pig Anti-Insulin (Dako, Glostrup, Denmark). The reaction was carried out in the Discovery XT System. Sections were incubated at 37 ^°^C with primary antibody (1:60) for 1 h in Ab diluent. The secondary antibody used was OmniMap anti-Rb HRP and detection was carried out with ChromoMap DAB. Contrast staining was performed with hematoxylin and bluing reagent. The relative insulin-positive area was determined on slides (n = 5 per group). The presence of vessels was confirmed by immunohistochemical CD-31 labelling using the Discovery XT System again. After deparaffination and rehydration, sections were placed in reaction buffer. Unmasking was performed in standard “CC1” conditions (60 min, 100 ^°^C). Sections were then incubated at 37 ^°^C with an anti-CD31 polyclonal antibody (Abcam, Cambridge, United Kingdom), 1:50 for 32 min. The secondary antibody used was OmniMap anti-Rb HRP and detection was carried out with ChromoMap DAB. Contrast staining was performed with hematoxylin and bluing reagent. Vessel numbers were determined by counting total CD-31 positive vessels in equivalent areas (6 fields per slide, n = 4 per group) observed on the matrix surrounding islets. Leukocyte infiltration throughout the islet graft was quantified by the number of myeloperoxidase (MPO) positive cells. The reaction was carried out with an anti-MPO antibody (Thermo, Fisher Scientific, Waltham, MA) also in the Discovery XT System. First, unmasking was performed by heating at pH 8.4 with CC1 buffer. Then, slides were blocked with the casein based Antibody block and incubated with the antibody at 37 ^°^C, for 1 h and at a 1:50 dilution in Ab diluent. The next steps were the same as those described for insulin. The number of MPO-positive cells in the equivalent areas was determined on slides (6 fields per slide, n=4 per group). All reagents, except where specified, were from Ventana, Oro Valley, AZ.

### Statistical analysis

Results are expressed as mean ± SEM. The significance of differences between two independent groups was calculated using the unpaired Student’s *t*-test. For multiple comparisons, ANOVA tests were conducted followed by the Scheffé *post hoc* test to determine specific differences between groups, unless otherwise indicated. A p-value <0.05 was considered statistically significant.

## Results

### Immunodeficient model: subcutaneous transplantation of PSI in diabetic mice

STZ-induced diabetic nude mice (n=5 per group) were transplanted subcutaneously with xenogenic islets alone (ISC) or embedded into a plasma-based scaffold without cells (PSI) or containing 2 x 10^5^ autologous fibroblasts (PSI-F), 2 x 10^5^ allogenic BM-MSCs (PSI-M) or 2 x 10^5^ of both autologous fibroblasts and allogenic BM-MSCs (PSI-FM).

**Figure 1:**
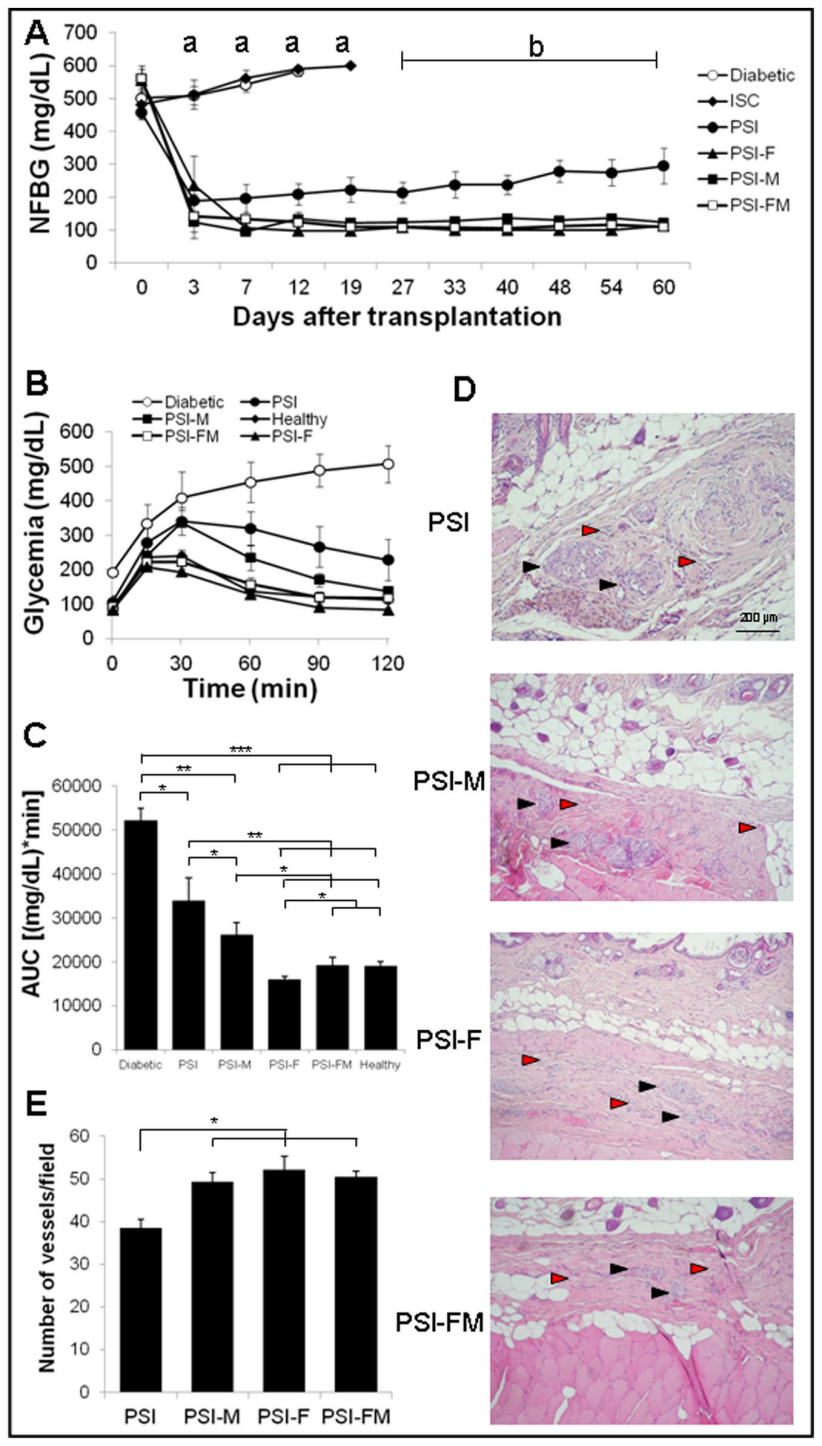
Subcutaneous transplantation of PSI in immunodeficient diabetic mice. A) Follow up of NFBG in the following groups: (○) non-transplanted mice; (♦) mice subcutaneously transplanted with free islets (ISC); (●) mice subcutaneously transplanted with PSI; (■) mice subcutaneously transplanted with PSI containing 2x10^5^ allogenic BM-MSCs (PSI-M); (▲) mice subcutaneously transplanted with PSI containing 2x10^5^ autologous fibroblasts (PSI-F); (□) mice subcutaneously transplanted with PSI containing 2x10^5^ of both autologous fibroblasts and allogenic BM-MSCs (PSI-FM). B) Blood glucose responses to IPGTTs, 60 days after transplantation. Groups are the same as detailed in A except for (◊) non-transplanted healthy mice. C) AUC data obtained from IPGTTs. D) Histology of subcutaneous islet xenografts 60 days after transplantation in hematoxylin and eosin stained samples. Black arrows point to islets and red arrows point to vessels. E) Quantification of vessels in the subcutaneous grafts after CD31 immunostaining. For all groups n=5. Statistical significances (p<0.05): (^a^) ISC and diabetic vs the other groups; PSI vs PSI-M, PSI-F and PSI-FM; (^b^) PSI vs PSI-M, PSI-F and PSI-FM. (*) p<0.05; (**) p<0.01.

None of the non-transplanted diabetic mice or mice transplanted subcutaneously with free islets (ISC) reversed hyperglycemia (Figure 1A). However, when islets were transplanted into PSI without cells, mice reversed hyperglycemia for the first days after transplantation, but were not able to maintain a stable normoglycemia, showing a progressive increase of NFBG in subsequent days. Nevertheless, the presence of fibroblasts (PSI-F), BM-MSCs (PSI-M) or both (PSI-FM) in the PSI allowed mice to achieve normoglycemia and to maintain it stably throughout the follow up, without significant differences between the three groups. The IPGTT performed 60 days after transplantation showed a non-physiological glucose tolerance response by PSI mice, although their function was significant better than that shown by non-transplanted diabetic mice (Figure 1B and C). Both PSI-F, PSI-M and PSI-FM mice showed a physiological-like response, with PSI-F and PSI-FM showing a significantly better function than PSI-M. Histological study showed insulin-positive islets completely integrated in a new-formed collagen-like matrix at the subcutaneous space of PSI, PSI-M, PSI-F and PSI-FM mice (Figure 1D). Nevertheless, the matrix appeared more organised and better integrated with adjacent tissues when fibroblasts, BM-MSCs or both where contained in the PSI. Vessel counting showed that the presence of fibroblasts, BM-MSCs or both in the PSI improved graft vascularization (Figure 1 E). No significant insulin staining was observed in the pancreata of any of the diabetic mice subjected to subcutaneous transplantation (data not shown).

### Immunocompetent model

#### Subcutaneous transplantation of PSI in diabetic mice

STZ-induced diabetic Luc[-] mice were transplanted subcutaneously with xenogenic islets alone (ISC, n=5) or embedded into a PSI without cells (PSI, n=5) or containing: autologous fibroblasts (PSI-F), allogenic BM-MSCs (PSI-M), or autologous fibroblasts + allogenic BM-MSCs (PSI-FM). In each group, three different numbers of cells were studied: 2 x 10^5^ (n=5), 4 x 10^5^ (n=5) and 10^6^ (n=7).

**Figure 2:**
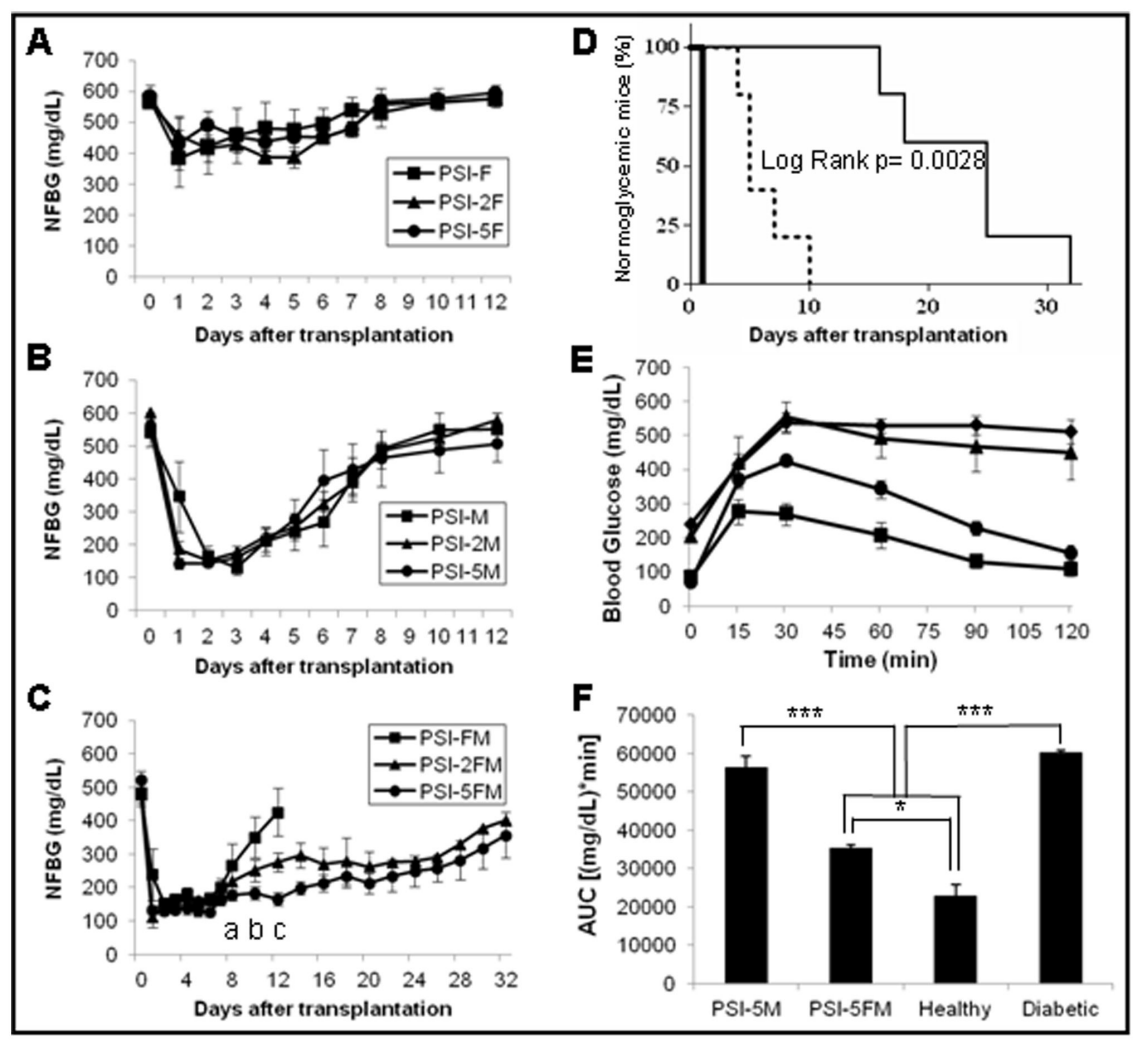
Subcutaneous transplantation of PSI in immunocompetent diabetic mice. A) Follow up of NFBG in mice subcutaneously transplanted with PSI containing three different cell doses of autologous fibroblasts: 2 x 10^5^ (PSI-F, n=5), 4 x 10^5^ (PSI-2F, n=5) and 1 x 10^6^ (PSI-5F, n=7). B) Follow up of NFBG in mice subcutaneously transplanted with PSI containing three different cell doses of allogenic BM-MSCs: 2 x 10^5^ (PSI-M, n=5), 4 x 10^5^ (PSI-2M, n=5) and 1 x 10^6^ (PSI-5M, n=7). C) Follow up of NFBG in mice transplanted with PSI containing three different cell doses of both autologous fibroblasts and allogenic BM-MSCs: 2 x 10^5^ (PSI-FM, n=5), 4 x 10^5^ (PSI-2FM, n=5) and 1 x 10^6^ (PSI-5FM, n=7). All results are expressed as mean ± SEM. Statistical significances (p<0.05): (^a^) PSI-5FM vs PSI-FM and PSI-2FM; (^b^) between the three groups; (^c^) PSI-2FM vs PSI-5FM. D) Percentage of animals with normoglycemia after transplantation. Thick line, PSI-5F; dotted line, PSI-5M; thin line, PSI-5FM. E) Blood glucose responses to IPGTTs performed 7 days after transplantation in the following groups of diabetic mice: (♦), non-transplanted; (▲), PSI-5M; (●), PSI-5FM; (■), non-transplanted healthy mice. For all groups n=7. F) AUC data obtained from IPGTTs. (*) p<0.05; (***) p<0.001.

None of ISC or PSI mice were able to reverse hyperglycemia (data not shown) not even when fibroblasts were added to PSI (PSI-F mice) (Figure 2A). However, when fibroblasts were substituted with BM-MSCs (PSI-M), hyperglycemia was reversed in the first two days after transplantation, although normoglycemia was only maintained for a mean period of 4 ± 1.1 days (Figure 2B). No cell dose-effect was observed in either PSI-F (Figure 2A) or PSI-M mice (Figure 2B). Interestingly, when both autologous fibroblasts and allogenic BM-MSCs were co-transplanted into PSI (PSI-FM), normoglycemia was also achieved in the first two days after transplantation but, in this case, maintained for a mean period of 7.2 ± 0.5 days (Figure 2C). Moreover, the increase of the number of both BM-MSCs and fibroblasts co-transplanted together in the PSI showed a clear cell-dose effect. By doubling the cell number of both cell types (PSI-2FM), normoglycemia was maintained 11.6 ± 3.04 days after transplantation (with one mouse maintaining normoglycemia until day 20), while when increasing 5-fold the cell number (PSI-5FM), normoglycemia was maintained 18.4 ± 3.86 days after transplantation (with one mouse maintaining normoglycemia until day 30) (Figure 2D and Table 1).

**Table 1:** Period of normoglycemia after subcutaneous transplantation of PSI in diabetic immunocompetent mice.

**Group**	**n**	**Days of normoglycemia**
PSI + 10^6^ BM-MSCs_alo_	7	4.2 ± 1.07^a^
PSI + 2x 10^5^ BM-MSCs_allo_ and 2 x 10^5^ fibroblasts_auto_	5	7.2 ± 0.49^b^
PSI + 4x 10^5^ BM-MSCs_allo_ and 4 x 10^5^ fibroblasts_auto_	5	11.6 ± 3.04
PSI + 10^6^ BM-MSCs_allo_ and 10^6^ fibroblasts_auto_	7	18.4 ± 3.86

^a^ p<0.05 versus all groups.

^b^ p<0.05 versus PSI + 10^6^ BM-MSCs_allo_ and 10^6^ fibroblasts_auto_

ANOVA of data followed by the Scheffé *post hoc* test

All results are expressed as mean ± SEM.

PSI, plasma-based scaffold containing xenogenic islets; BM-MSCs_allo_, allogenic bone marrow-mesenchymal stem cells; fibroblasts_auto_, autologous skin fibroblasts

To further study the functional efficacy of the islet xenografts, IPGTTs were performed in the three groups transplanted with the highest dose of cells (PSI-5F, PSI-5M and PSI-5FM), seven days after transplantation. Day seven was chosen since maximum NFBG differences within these groups were observed. IPGTT for PSI-5F mice was similar to control diabetic mice (data not shown), in accordance with their high NFBG throughout the study. In the same way, mice transplanted with PSI containing BM-MSCs (PSI-5M) showed a diabetic-like response, in spite of their lower NFBG when compared to that of PSI-5F mice (Figure 2E). Mice transplanted with both BM-MSCs and fibroblasts (PSI-5FM) showed a physiological-like response, with a blood glucose tolerance significantly better than PSI-5M (Figure 2F). In spite of a good glucose tolerance, a significant difference was also observed when comparing PSI-5FM with healthy control mice.

#### In vivo follow-up of cells transplanted subcutaneously in the PSI

**Figure 3:**
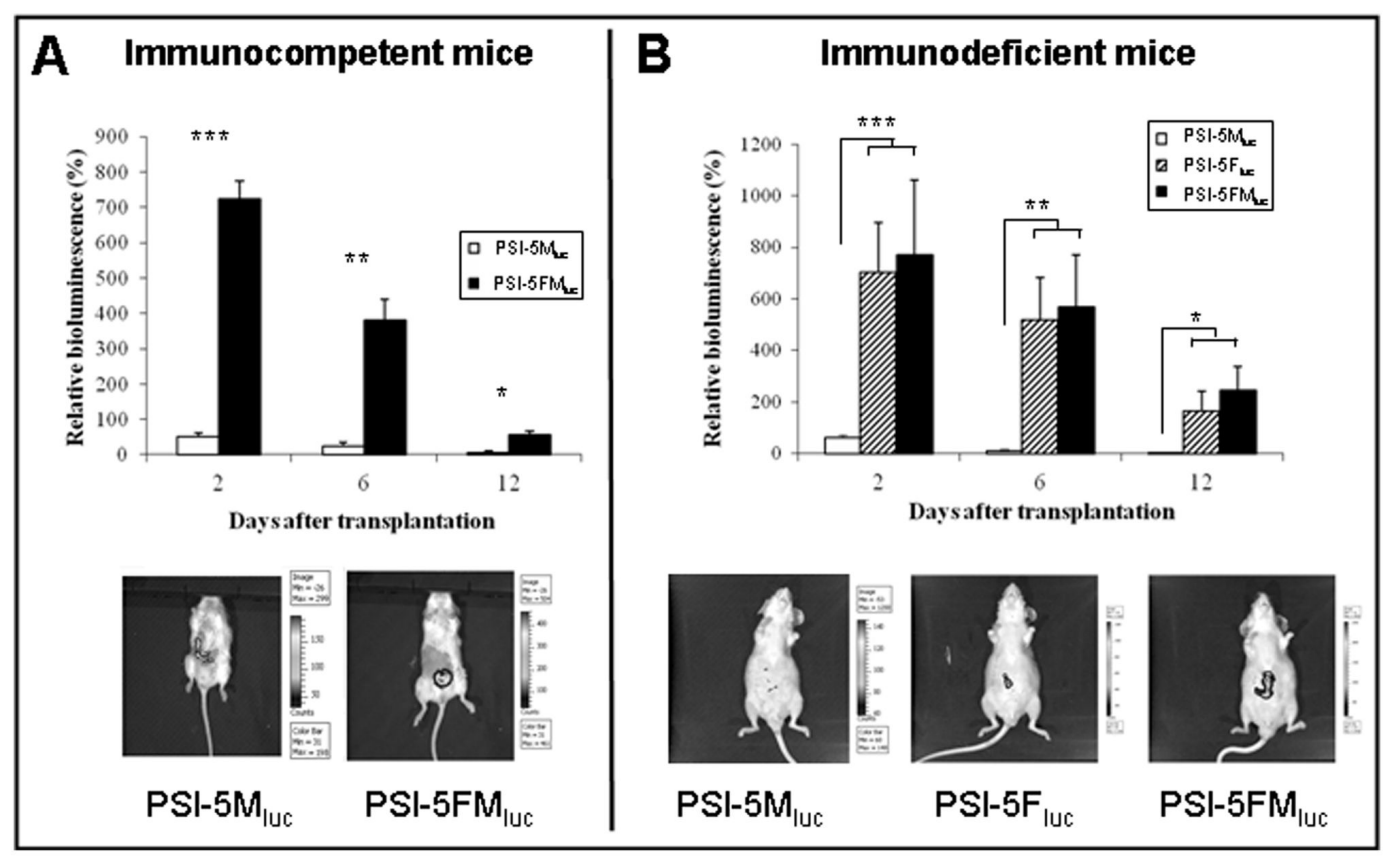
Evolution of bioluminescent cells embedded in PSI and subcutaneously transplanted in both immunocompetent and immunodeficient mice. A) *Immunocompetent mice*. Relative luminescence signal of 10^6^ allogenic Luc[+]-BM-MSCs transplanted alone into PSI (PSI-5M_luc_) or co-transplanted with 10^6^ autologous Luc[-]-fibroblasts (PSI-5FM_luc_), as well as representative IVIS® lumina images of previously described mice, 12 days after transplantation. B) *Immunodeficient mice*. Relative luminescence signal of 10^6^ allogenic Luc[+]-BM-MSCs transplanted alone into PSI (PSI-M_luc_) or co-transplanted with 10^6^ autologous Luc[-]-fibroblasts (PSI-FM_luc_), as well as luminescence signal of 10^6^ allogenic Luc[+]-fibroblasts transplanted alone into PSI (PSI-F_luc_). Representative IVIS® lumina images of previously described mice, 12 days after transplantation. Values related to day 0 (100%) are represented as mean ± SEM. For all groups n=5. (*) p<0.05; (**) p<0.01; (***) p<0.001.

Behaviour and viability of allogenic Luc[+]-BM-MSCs embedded in PSI and transplanted subcutaneously in immunocompetent mice (n=5 per group), was addressed by determining *in vivo* bioluminescence. The bioluminescence of BM-MSCs transplanted alone in PSI (PSI-5M_luc_) showed a marked decrease after transplantation, with only 6.2% remaining at day 12 (Figure 3A). In contrast, bioluminescence of BM-MSCs subcutaneously co-transplanted with autologous Luc[-]-fibroblasts in PSI (PSI-5FM_luc_), increased markedly 2 days after transplantation, and decreased during subsequent days. In spite of this, the bioluminescence of BM-MSCs co-transplanted with fibroblasts was always significantly higher than that of BM-MSCs transplanted alone.

To check whether a possible immunological effect could explain the reduced survival of allogenic BM-MSCs, subcutaneous transplantation was performed in immunodeficient mice (n=5 per group). Bioluminescence of Luc[+]-BM-MSCs transplanted alone in PSI (PSI-5M_luc_) also showed a marked decrease, with 4.27% remaining at day 12 (Figure 3B). When co-transplanted with autologous Luc[-]-fibroblasts (PSI-5FM_luc_), a significant increase of BM-MSC bioluminescence was observed at day 2 after transplantation, which decreased in the following days. Again, bioluminescence was still significantly higher than that of BM-MSCs transplanted alone (PSI-5M_luc_). Interestingly, allogenic Luc[+]-fibroblasts transplanted alone in PSI (PSI-5F_luc_) showed a bioluminescence evolution very similar to that shown by allogenic Luc[+]-BM-MSCs when co-transplanted with autologous Luc[-]-fibroblasts (PSI-5FM_luc_).

#### Histological examination of subcutaneous islet xenografts

**Figure 4:**
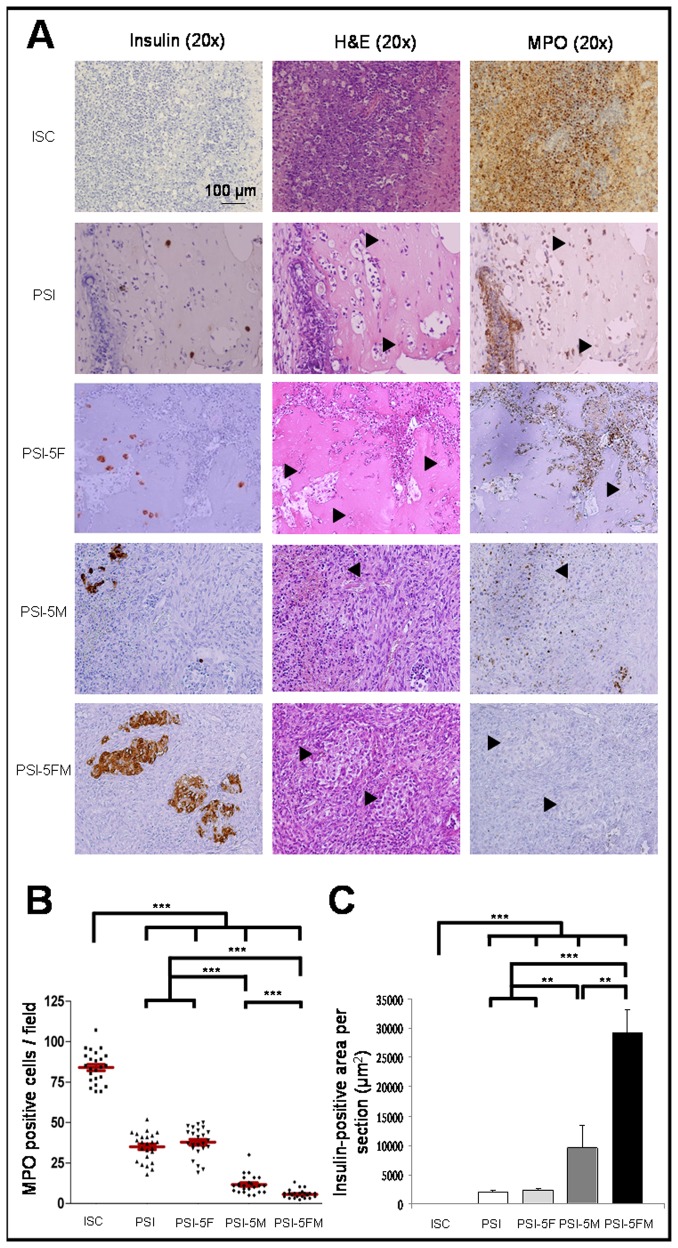
Histology of subcutaneous islet xenografts in immunocompetent diabetic mice. A) Representative images of subcutaneously transplanted islets alone (ISC), PSI without cells and PSI containing: 10^6^ autologous fibroblasts (PSI-5F), 10^6^ allogenic BM-MSCs (PSI-5M), or 10^6^ of both autologous fibroblasts and allogenic BM-MSCs (PSI-5FM). Islet grafts were retrieved seven days after transplantation in diabetic mice. Samples were stained with H and E, or labelled with anti-insulin or anti-MPO antibodies. Black arrows point to islets. B) Quantification of the number of MPO-positive cells per field present on the leukocyte infiltration of the islet graft. C) Quantification of the insulin-positive area per section present on the islet graft. (**) p<0.01; (***) p<0.001.

Histological analysis of subcutaneous islet xenografts was performed seven days after transplantation in diabetic immunocompetent mice (Figure 4). Xenografts of ISC, PSI without cells or containing autologous fibroblasts (PSI-5F) showed a great lymphocyte and polymorphonuclear cells (PMN) infiltration. No insulin-positive cells were observed in ISC grafts, while PSI and PSI-5F grafts showed small aggregates of insulin-positive cells, resulting from islet degradation, surrounded by non-integrated plasma scaffold. Xenografts containing allogenic BM-MSCs (PSI-5M) showed less lymphocyte infiltration with a lower number of PMNs. Some insulin-positive islets were observed, but most of them showed symptoms of injury like disrupted insulin staining and irregular morphology. Finally, xenografts containing autologous fibroblasts and allogenic BM-MSCs (PSI-5FM) showed the least lymphocyte and PMN infiltration. Furthermore, several insulin-positive islets were observed, most of them with normal morphology and continuous insulin staining.

Additionally, we quantified the extension of leukocyte infiltration by MPO-staining, a valid measure for the extent of organ rejection [30]. The number of MPO positive cells present in the PSI-5M xenografts was significantly lower with respect to that of PSI-F, PSI and ISC mice, and it was even lower in those transplanted with both BM-MSCs and fibroblasts (PSI-5FM) (Figure 4B). Likewise, we measured the presence of insulin-positive areas, which indirectly correlated with MPO-staining, indicating a higher-maintained islet function when immunological response was lower (Figure 4C).

## Discussion

Two of the main key points of islet transplantation are the site of engraftment and the control of the immune response. The subcutaneous space could be a good alternative, but its main limitation resides in its poor vascularization. In this sense the pro-angiogenic and immunomodulatory properties of different cell types, like dermal fibroblasts [22,31] or MSCs [17,18,32], have been described. The work presented here shows how islets embedded in a plasma-based scaffold (PSI) transplanted subcutaneously were able to reverse hyperglycemia in diabetic immunodeficient mice, although the presence of BM-MSCs and/or fibroblasts in the graft was a requirement for maintaining long-term islet function. Nevertheless, in immunocompetent mice, the presence of fibroblasts in PSI did not reverse hyperglycemia, while BM-MSC presence allowed its reversal in all transplanted mice for a short period of time. Surprisingly, when both cell types were co-transplanted together into the PSI, a better control of glycemia was observed, achieving up to one month of normoglycemia without using immunosuppressive drugs.

Previous reports have shown that fibrin gel, in addition to its pro-angiogenic properties, supports cell survival, growth and differentiation through the intermediary function of specific extracellular matrix proteins [33–35]. Fibrin has also been used to maintain the three-dimensional configuration of human islets *in vitro*, giving rise to a robust functional graft under the kidney capsule of nude mice [36], and as a carrier for islet transplantation [37]. In the present work, we have extended these results demonstrating that subcutaneous transplantation of islets in diabetic immunodeficient mice reversed hyperglycemia when embedded in a plasma gel (PSI), suggesting an active participation of this scaffold as extracellular matrix [38]. Similarly, as occurred in several studies in immunodeficient or syngenic models [25,39–41], the presence of fibroblasts and/or BM-MSCs improved islet function and viability due to their pro-angiogenic properties, favouring graft revascularization and normoglycemia restoration.

Regarding islet transplantation in immunocompetent recipients, only allogenic BM-MSCs exerted an immunomodulatory effect at the subcutaneous space. Nevertheless, this positive effect was only partial towards glycaemic normalization. A plausible explanation for this result could be the reduced survival of these cells during the first days after transplantation, which was observed both in immunocompetent and immunodeficient mice, pointing to a non-immunological cause as responsible for this effect. In a poor vascularized subcutaneous space, it is feasible that sparse blood supply and low oxygen tension may lead to BM-MSC loss as has been described under hypoxic conditions and serum deprivation [42]. In our model, fibroblasts improved islet and BM-MSCs survival, underlining their pro-angiogenic effect on graft revascularization and, therefore, reduction of hypoxia [43]. Unlike BM-MSCs, it has been described that fibroblasts are cells with a higher tolerance to hypoxia which indeed have the potential to rapidly proliferate and migrate under hypoxic conditions, modulating their function to adapt vascular needs [44,45]. Furthermore, ischemic conditions induce an overexpression of the pro-angiogenic Hypoxia Inducible Factor-1 alpha and the pro-survival B-cell lymphoma 2 in fibroblasts [44,46]. Moreover, some factors secreted by these cells have been shown to stimulate BM-MSC proliferation [47], as well as to induce an increase in their survival [48,49]. However, in spite of the MSC-like immunosuppressive properties attributed to fibroblasts in the literature [50], mainly demonstrated by *in vitro* studies, no significant immunomodulatory effect was observed in our model.

Further studies are needed in order to address the underlying mechanisms of this cooperation. In this sense, we are currently performing *in vitro* studies to compare the “secretome” of BM-MSCs and Fibroblasts cultured alone or together, with or without the presence of islets, under hypoxic or normoxic conditions. Knowing the molecular mechanisms of this cooperative relationship among fibroblasts, MSCs and islets, is a required step in order to perform *in vivo* experiments to optimize the timing, dose, administration route and combination of cells and/or different factors in our model.

In this work, the evolution of fibroblasts and BM-MSCs contained in PSI has been analyzed by luminescent tracking, which is a commonly used strategy in *in vivo* cell survival and proliferation studies [51]. Luminescent data provide useful information about cell tracing and viability without sacrificing animals, but must be interpreted carefully since several factors, such as graft re-vascularization, must been taking into account due to their influence on the results, especially in the first days after transplantation [52]. Keeping this in mind, our data shows for the first time a positive effect of fibroblasts over BM-MSC proliferation and survival under such restricted conditions as in the subcutaneous space. As a result, BM-MSCs prolonged their effect in our system and thus, xenograft viability in the immunocompetent recipient.

The immunomodulatory properties of MSCs when co-transplanted with islets have been shown in several studies using well-vascularized sites for graft transplantation [53–55], where MSC survival may be higher, exerting their protective effect over longer periods and, therefore, improving graft viability. Moreover, most of them used allogenic models, usually under low-dose immunosuppressive therapy during the first days after transplantation. In this work we have shown the results of subcutaneous islet transplantation in a xenogenic model, without using any immunosuppressive drug throughout the study. Local or systemic administration of successive doses of BM-MSCs and/or short-term immunosuppression after islet transplantation, could improve our results. In addition, other cell types with pro-angiogenic and/or immunomodulatory properties such as adipose mesenchymal stem cells [56], sertoli cells [57] and/or endothelial cells [58], could be incorporated into the graft in order to optimize this model.

Development of autologous insulin-producing cells will surely become a reality in the future [59], avoiding the use of non-homologous pancreatic islets. Nevertheless, in spite of their autologous origin, and taking into account the immunological basis of type 1 diabetes, the use of immunoregulatory cells could be a necessary strategy. The use of a bioartificial endocrine pancreas could be the definitive solution to type 1 diabetes [60], be it by development of “insulin pumps” which respond automatically to blood glucose levels or be it by development of tissue-engineered pancreas. In this work, we have developed a subcutaneous “endocrine pancreas” by tissue engineering which is able to work physiologically in immunodeficient mice and, at least temporarily, in immunocompetent ones. The development of tissue engineered models, like the PSI, could be a successful strategy to develop an “artificial endocrine pancreas” by combining pancreatic islets, or maybe insulin-producing cells, with other cell types which improve graft revascularization and modulate the immune response, achieving a physiological-like endocrine pancreas in an ectopic site without using immunosuppressive drugs. This system could become a successful strategy in the field of islet transplantation in the future.
